# Trans-sodium crocetinate suppresses apoptotic and oxidative response following myoglobin-induced cytotoxicity in HEK-293 cells

**DOI:** 10.22038/IJBMS.2024.75306.16322

**Published:** 2024

**Authors:** Tahereh Aminifard, Soghra Mehri, Mahboobeh Ghasemzadeh Rahbardar, Fatemeh Rajabian, Abolfazl Khajavi Rad, Hossein Hosseinzadeh

**Affiliations:** 1 Department of Pharmacodynamics and Toxicology, School of Pharmacy, Mashhad University of Medical Sciences, Mashhad, Iran; 2 Pharmaceutical Research Center, Pharmaceutical Technology Institute, Mashhad University of Medical Sciences, Mashhad, Iran; 3 Department of Physiology, School of Medicine, Mashhad University of Medical Sciences, Mashhad, Iran

**Keywords:** Apoptosis, Autophagy, Acute Kidney injuries, Myoglobin, Rabdomyolysis, Trans-sodium crocetinate

## Abstract

**Objective(s):**

Rhabdomyolysis (RM) is a serious fatal syndrome. The RM leads to acute kidney injury (AKI) as a fatal complication. The belief is that RM-induced AKI is triggered by myoglobin (MB). MB activates oxidative and apoptotic pathways. Trans-sodium crocetinate (TSC) is obtained from saffron. It has anti-oxidant and renoprotective effects. This research was designed to assess the mechanisms of MB-induced cytotoxicity in HEK-293 cells (human embryonic kidney cells) as well as the possible effects of TSC against MB-induced cytotoxicity.

**Materials and Methods:**

HEK-293 cells were exposed to diverse concentrations of TSC (2.5, 5, 10, 20, 40, 80, and 100 µM) for 24 hr. Then, MB (9 mg/ml) was added to the cells. After 24 hr, cell viability was measured through MTT, and the values of ROS generation were calculated using DCFH-DA assay. Also, autophagy and apoptosis markers in cells were assessed by western blot analysis.

**Results:**

MB decreased viability and increased ROS levels in HEK-293 cells. However, pretreatment of HEK-293 cells with TSC for 24 hr reduced the cytotoxicity and ROS production caused by MB. Furthermore, MB enhanced both the apoptosis (cleaved caspase-3 and Bax/Bcl-2 ratio) and autophagy markers (LC3II/I ratio and Beclin-1) in HEK-293 cells. On the other hand, TSC pretreatment condensed the levels of autophagy and apoptosis criteria in response to MB cytotoxicity.

**Conclusion:**

TSC has a positive effect in preventing MB-induced cytotoxicity in HEK-293 cells by increasing anti-oxidant activity and regulation of apoptotic and autophagy signaling pathways.

## Introduction

Rhabdomyolysis (RM) is a medical disorder characterized by damage to skeletal muscle fibers with the distribution of their whole contents to the bloodstream. Myoglobin (MB), lactate dehydrogenase, and creatine phosphokinase are the greatest important substances released into the blood circulation (1). Acute kidney injury (AKI) is an important and known complication of RM. MB induces intrarenal vasoconstriction, tubular obstruction, and direct tubule injury which are mainly involved in RM-induced AKI (2).

RM-induced AKI is triggered by MB which induces renal dysfunction (3). MB is an iron and oxygen-binding protein (4). MB significantly releases from myocytes when skeletal muscle fibers distribute and RM occurs. A clinical study demonstrated that renal uptake of MB is mediated via endocytic receptors (5). MB is one of the most significant toxic factors that causes renal cell injury through direct and indirect mechanisms (6).

 MB promotes reactive oxygen species (ROS) generation and redox-active iron generation, apoptosis, and inflammation (3). The permeability of the mitochondrial membrane can be changed by MB, which consequently induces the secretion of cytochrome c, caspase-3 activation, and renal tubular cell apoptosis. The former investigation suggested that, besides apoptosis, other forms of cell death may be involved in MB-induced cytotoxicity (4). Taken together, results confirmed that MB-induced oxidative damage is a significant cause of renal cell apoptosis in RM-induced AKI (7).

The underlying mechanisms of RM-induced AKI have been studied extensively in *in vivo* models of AKI induced by glycerol in rats. The most important pathophysiological mechanisms are intraluminal cast production, renal vasoconstriction, and MB-induced cytotoxicity. On the other hand, *in vitro* studies are conducted to investigate the direct cytotoxic effects of MB on renal cells, as there are no intervening factors such as hypoxia after vasoconstriction that occur *in vivo*. These research projects suggest that direct MB-induced cytotoxicity has an important role in the progression of RM-induced AKI via evoking oxidative stress and inflammatory and apoptotic responses (7). *In vivo* studies of AKI and *in vitro* models of MB-induced cytotoxicity in HEK-293 cells (human embryonic kidney cells) allow researchers to investigate the underlying mechanisms of RM-induced AKI and to test potential therapeutic interventions (3). 

The occurrence of RM-induced AKI has enhanced over the prior decade, so, in recent years, research on useful therapies to prevent RM-induced AKI has attracted much attention (1). Herbal therapy plays a significant role and is a crucial aspect of healthcare. This could be due to cultural acceptability, safety, potency, and fewer side effects (8). Saffron is a traditional and well-known herb that belongs to the Iridaceae family (9) and has a long history in traditional medicine (10). Crocetin is an ingredient of saffron (11), which exhibits various pharmacological attributes such as hepatoprotective (12), neuroprotective (13), cardioprotective (14), and anti-atherosclerosis (15). The renoprotection property of crocetin, can be mediated through anti-oxidant (16), anti-inflammatory (17), anti-apoptotic (18), and autophagy regulation (19) mechanisms. Crocetin normalized intracellular Ca^2+^ homeostasis, scavenged intracellular ROS, and raised anti-oxidant protection in bovine aortic endothelial cells (20). It prevented inflammatory cytokine production, elevated anti-inflammatory cytokines in plasma, and inhibited nuclear factor kappa-light-chain-enhancer of activated B cells (NF-κB) activation in APPsw transgenic mice because crocetin has a pseudo-nonsteroidal anti-inflammatory drug (NSAID) character (21). Crocetin suppressed extracellular signalregulated protein kinase (ERK1/2), phosphoinositide 3-kinases (PI3K) /AKT, and p38 expression in KYSE-150 cells and consequently inhibited mitochondrial-mediated apoptosis (22). Also, in HEK-293 cells, crocetin repaired the anti-oxidant system and prevented ROS production. Likewise, it prevented ROS release from mitochondria into the cytoplasm through the steadiness of the mitochondrial membrane potential (23). Numerous lines of documents reported the advantageous effects of crocetin as a potent anti-oxidant in the protection of kidney disorders (24). Therefore, crocetin can be a useful remedy for MB-induced cytotoxicity.

Trans-sodium crocetinate (TSC) is a popular trans-carotenoid salt, which is obtained from crocetin (25). TSC increases the oxygen transfer from erythrocytes to tissues and oxygen diffusivity and decreases plasma resistance (26). So it has been developed to enhance the delivery of oxygen (O_2_) to damaged and hypoxic tissues (27). Additionally, TSC can enhance the effects of crocetin by improving its bioavailability (28). Moreover, TSC as an anti-oxidant has a protective effect against renal injury in multiple reports (26).

Thus, the current study was undertaken to investigate the potential nephroprotective effect of TSC against MB-induced cytotoxicity in HEK-293 cells. Moreover, the underlying cellular signaling pathways for the anti-apoptotic, autophagy regulation, and oxidative stress effects of TSC on MB-induced cytotoxicity in HEK-293 cells were evaluated.

## Materials and Methods

***Chemicals***

MB from equine skeletal muscle was obtained from Sigma (Munich, Germany). TSC and vitamin C (Vit C) were bought from Tinab Shimi (Mashhad, Iran). Fetal bovine serum (FBS) and Dulbecco’s modified Eagle’s medium (DMEM) high glucose were procured from Gibco (New York, USA). Fluorescent probe 2, 7-dichlorofluorescein diacetate (DCF-DA) and 3-(4,5-dimethylthiazol-2-yl)-2, 5-diphenyl tetrazolium (MTT) were bought from Sigma (Munich, Germany). The polyvinylidene fluoride (PVDF) membrane was procured from Bio-Rad (CA, USA). 

***Cell culture studies***

In the present research, HEK-293 cells were purchased from the Pasteur Institute (Tehran, Iran). In summary, HEK-293 cells were cultured in DMEM complemented with streptomycin (100 µg/ml), penicillin (100 U/ml), and heat-inactivated FBS (10% v/v). HEK-293 cells were preserved in a humidified atmosphere (90%) at 37 °C containing 5% CO_2_ in the air (29).

The experimental protocols were conducted under the guidelines of the Ethics Committee at the Mashhad University of Medical Sciences (Ethical Number: IR.MUMS.PHARMACY.REC.1399.033).

***MB-induced cytotoxicity***

MB was prepared in the reduced condition by dissolving 356 mg MB and 32.5 mg Vit C in DMEM (100 ml). The solution was blended and filtered via a 0.22-μm filter (7). In the MB-induced cytotoxicity group, HEK-293 cells were cultured for 24 hr and then incubated for another 24 hr with DMEM containing reduced MB. In the crocetin intervention group, at first, cells were pre-treated with diverse concentrations of TSC (2.5, 5, 10, 20, 40, 80, and 100 µM) for 24 hr and then exposed to MB. 

***Cell viability assay***

The viability of HEK-293 cells was surveyed via the MTT test in 96-well microplates (2500 cells/well). To assess the cytotoxic effect of MB on cells, the attached HEK-293 cells were incubated in DMEM at 37 °C with several concentrations of MB (0-15 mg/ml) for 24 hr, and cell viability was determined by the MTT test. The GraphPad Prism 8 statistical software was used to evaluate the concentration of MB that leads to 50% cell death (IC_50_). To investigate the cytoprotective effect of TSC, HEK-293 cells were incubated at 37 °C in DMEM for 24 hr. Afterward, various nontoxic concentrations of TSC (2.5, 5, 10, 20, 40, 80, and 100 µM) were added and the plate was incubated for 24 hr. Then, MB at a concentration of 9 mg/ml was added and incubated for the next 24 hr. Finally, the cells were treated with MTT solution (0.5 mg/ml), and incubated at 37 °C. After 2 hr the medium was eliminated and 100 µl dimethylsulfoxide (DMSO) was added individually to each well and shaken in the dark and at room temperature for up to 15 min for solving purple formazan crystals. Using an ELISA reader (Start Fax-2100, UK) the absorbance was measured at the wavelength of 545 and 630 nm.

***Measurement of intracellular ROS generation***

The accumulation of intracellular ROS was determined by the fluorometric technique (DCFH-DA assay) (30). HEK-293 cells (2500 cells/well) were cultured in plates (96-well) and incubated for 24 hr. The cells were pre-treated with different concentrations of TSC (1, 2.5, 5, 10, 20, 40, 80, and 100 µM) for 24 hr. Afterward, MB (9 mg/ml) was added and incubated for another 24 hr. Following incubation, cells were washed with PBS 2 times and then incubated at 37 °C with DCFH-DA (10 µM) for 30 min. The fluorescence intensity of DCF was assessed at 485 nm (excitation wavelength) and 528 nm (emission wavelength).

***Western blot analysis***

The expression of autophagy proteins and apoptotic pathway proteins were determined through the western blot technique in HEK-293 cells. Following the exposure of HEK-293 cells to TSC (40 µM) and MB (9 mg/ml), the cell pellets were collected and homogenized in the lysis buffer containing 50 mM Tris-HCl (pH: 7.4), 2 mM EDTA, complete protease inhibitor cocktail, 1 mM sodium orthovanadate (Na_3_VO_4_), 10 mM NaF, 2 mM EGTA, 10 mM beta-glycerophosphate, 1 mM phenylmethylsulfonyl fluoride (PMSF), and 0.2% W/V sodium deoxycholate. The homogenized samples were sonicated for 40 sec on ice via a probe sonicator (UP100H, Germany) to destroy the cell membrane and then centrifuged at 10,000 rpm for 10 min at 4 °C. The Bradford protein assay was done to determine the concentrations of protein in the supernatants. Homogenate extracts were blended with loading buffer (9:1 v/v; glycerol 20% v/v, Tris-base 100 mM, SDS 20% w/v, 2-ME 10% v/v, and bromophenol blue 0.2% w/v) and subsequently incubated in boiling water for 5 min. The samples were then aliquoted and kept at −80 °C. Supernatants with equal contents of total proteins were loaded and electrophoresed in 12% SDS-PAGE gels and transferred to PVDF membranes. Each membrane was individually blocked for 2 hr using Tris-buffered saline with tween 20% (TBST) containing 5% dry skim milk at room temperature. Following that, the membranes were incubated on a rocker with primary antibodies at 1:1000 dilutions, against Beclin-1 (Cell Signaling, #3495), LC3 II/I (Cell Signaling, #12741), cleaved caspase-3 (Cell Signaling, #9664), Bcl-2 (Cell Signaling, #2870), Bax (Cell Signaling, #2772), and mouse beta-actin (Cell Signaling, #3700) overnight at 4 °C. Afterwards the membranes were washed and incubated with anti-rabbit IgG labeled with horseradish peroxidase (Cell signaling, #7074) or anti-mouse IgG labeled with the enzyme (Cell signaling, #7076) at 1:3000 dilutions with gentle shaking for 2 hr at room temperature. Lastly, the optical densities of the bands were quantified by Alliance 4.7 Geldoc (UK). UVtec software (UK) was used for the densitometric analysis of the protein bands. In each membrane, protein bands were normalized relative to their β-actin protein as control (31).

***Statistical analysis***

The data are presented as mean ± standard deviation (SD). One-way ANOVA for statistical analyses and then the Tukey-Kramer post-test to compare means were used (Prism 8.0, GraphPad Software Inc., CA, USA). Differences were considered statistically significant when P was less than 0.05. The IC_50_ values were evaluated using Prism 8.0 (GraphPad Software Inc., CA, USA).

## Results

***Effect of MB on cell viability in HEK-293 cells***

**Figure 1:**
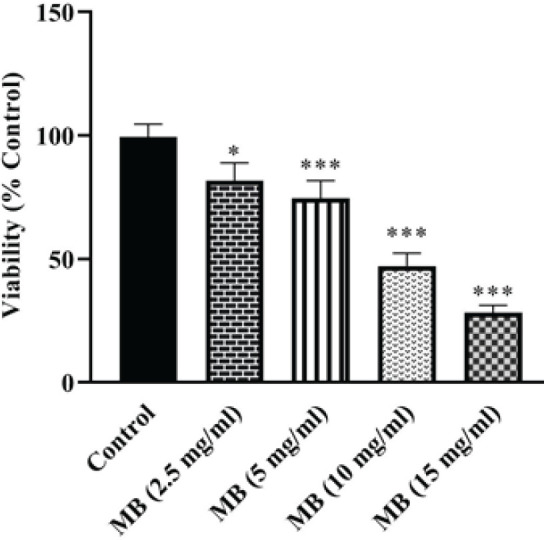
Effect of MB on HEK-293 cell viability evaluated by MTT assay. Data are presented as mean±SD of four separate experiments. Data were analyzed by one-way ANOVA and the Tukey-Kramer post-test. ***P<0.001 and*P<0.05 vs control-treated cells

Using the MTT test, the cytotoxicity of MB in HEK-293 cells was assessed. In comparison to the control group, HEK-293 cell viability exposed to MB for 24 hr was diminished in a concentration-dependent manner. The IC_50_ value of MB following 24 hr exposure was 9.19 ± 0.02 mg/ml (Figure 1).

***Effect of TSC on cell viability in HEK-293 cells***

**Figure 2:**
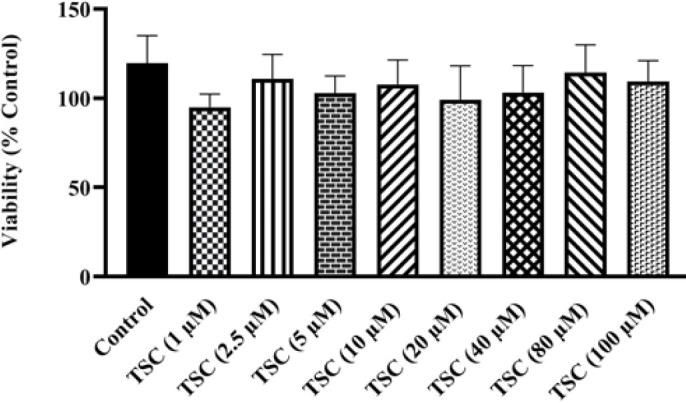
Effect of TSC on HEK-293 cell viability evaluated by MTT assay. Data are presented as mean±SD of four separate experiments. Data were analyzed by one-way ANOVA

TSC in diverse concentrations (0-100 µM) after 48 hr exposure had no significant toxic effect on HEK-293 cells in comparison to the control group (Figure 2). 

***Effect of TSC on MB-induced cytotoxicity in HEK-293 cells***

**Figure 3:**
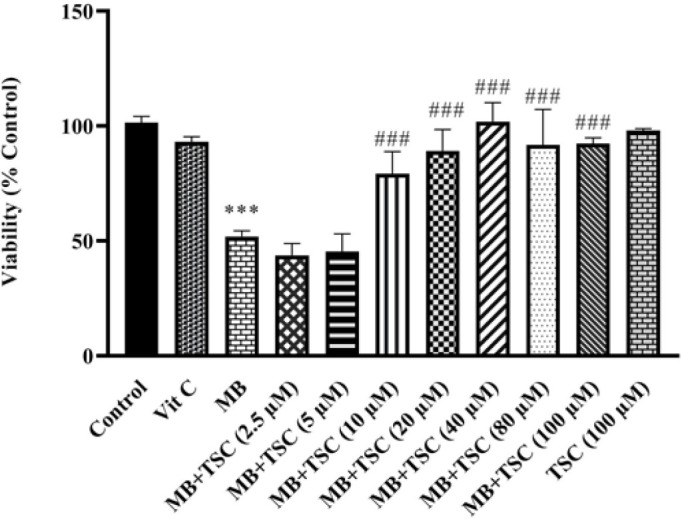
Effect of TSC on MB-induced toxicity on HEK-293 cells evaluated by MTT assay. Data are presented as mean±SD of four separate experiments. Data were analyzed by one-way ANOVA and the Tukey-Kramer post-test. ***P<0.001 vs control group, ###*P*<0.001 vs MB (9 mg/ml) treated cells

MB: Myoglobin; TSC: Trans-sodium crocetinate

The viability of HEK-293 cells exposed to MB (9 mg/ml) for 24 hr was meaningfully reduced compared to the control group (*P*<0.001). However, pretreatment of HEK-293 cells with TSC (2.5, 5, 10, 20, 40, 80, and 100 µM) for 24 hr meaningfully diminished cytotoxicity in comparison to the MB group (*P*<0.001) (Figure 3). Vit C had no significant toxic effect on HEK-293 cells. 

***Effect of TSC on MB-induced ROS generation in HEK-293 cells***

**Figure 4:**
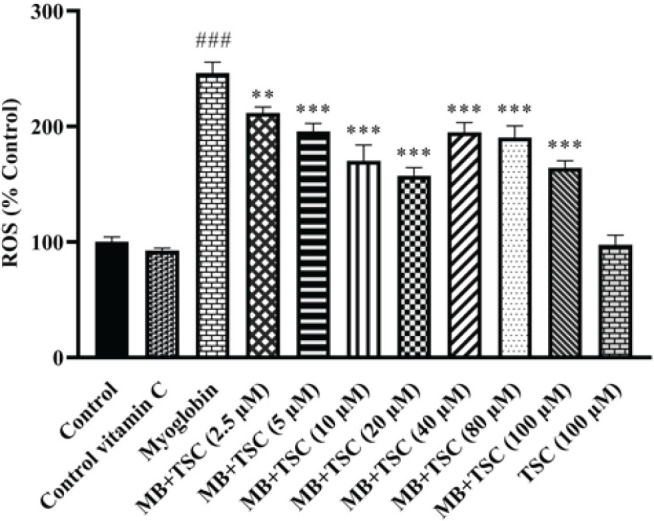
Effect of TSC on MB-induced ROS generation in HEK-293 cells. Data are presented as mean±SD of three separate experiments. Data were analyzed using one-way ANOVA and the Tukey-Kramer post-test. ###*P*<0.001 vs control group and ***P<0.001 and **P<0.01 vs MB (9 mg/ml) group

MB: Myoglobin; TSC: Trans-sodium crocetinate

Treatment of HEK-293 cells with MB at a concentration of 9 mg/ml for 24 hr significantly elevated intracellular ROS content in comparison to the control group (*P*<0.001). However, pretreatment with TSC at different concentrations (2.5, 5, 10, 20, 40, 80, and 100 µM) suppressed the production of ROS levels compared to the MB group (*P*<0.001). Vit C did not enhance the levels of ROS in contrast with the control group (Figure 4).

***Effect of MB and TSC on apoptotic factor in HEK-293 cells***

**Figure 5:**
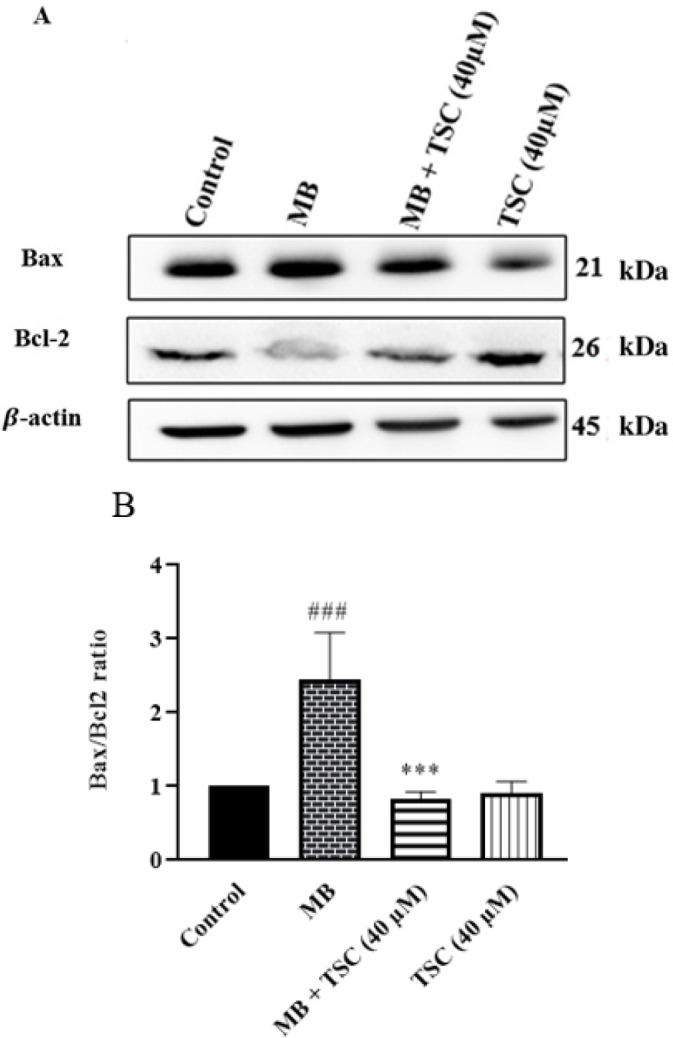
Effect of MB and TSC on Bax/Bcl-2 ratio in HEK-293 cells

**Figure 6:**
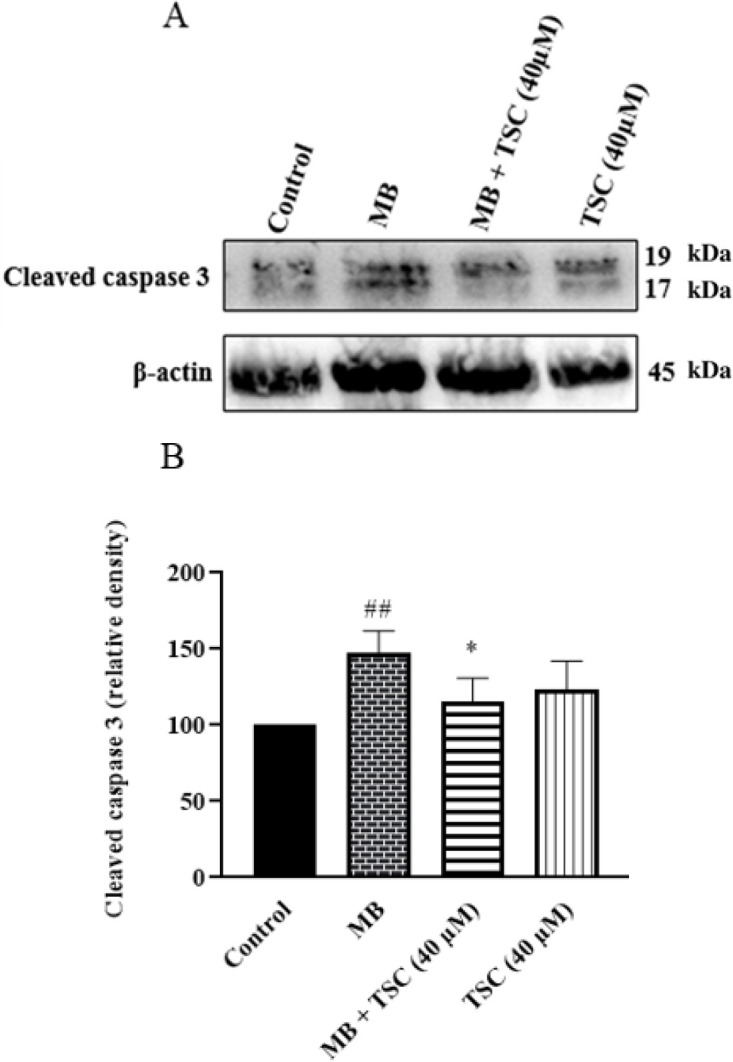
Effect of MB and TSC on cleaved caspase-3 expression in HEK-293 cells

To measure the expression of an apoptotic factor in HEK-293 cells, western blotting assay was done. According to Figures 5 and 6, the exposure of HEK-293 cells to MB (9 mg/ml) for 24 hr meaningfully increased cleaved caspase-3 (*P*<0.01 vs control) and the Bax/Bcl-2 ratio (*P*<0.001 vs control). On the other hand, in cells exposed to MB, pretreatment with TSC (40 μM, for 24 hr) decreased cleaved caspase-3 (*P*<0.05) and the Bax/Bcl-2 ratio (*P*<0.001) in comparison to the MB group.

***Effect of MB and TSC on autophagy proteins in HEK-293 cells***

**Figure 7:**
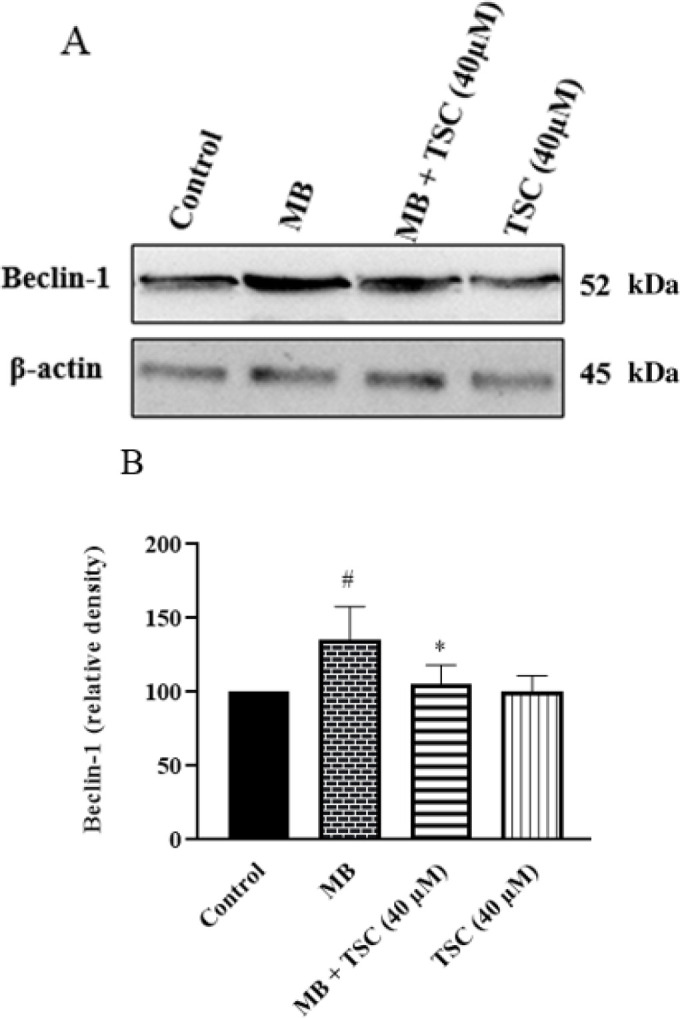
Effect of MB and TSC on Beclin-1 expression in HEK-293 cells

**Figure 8:**
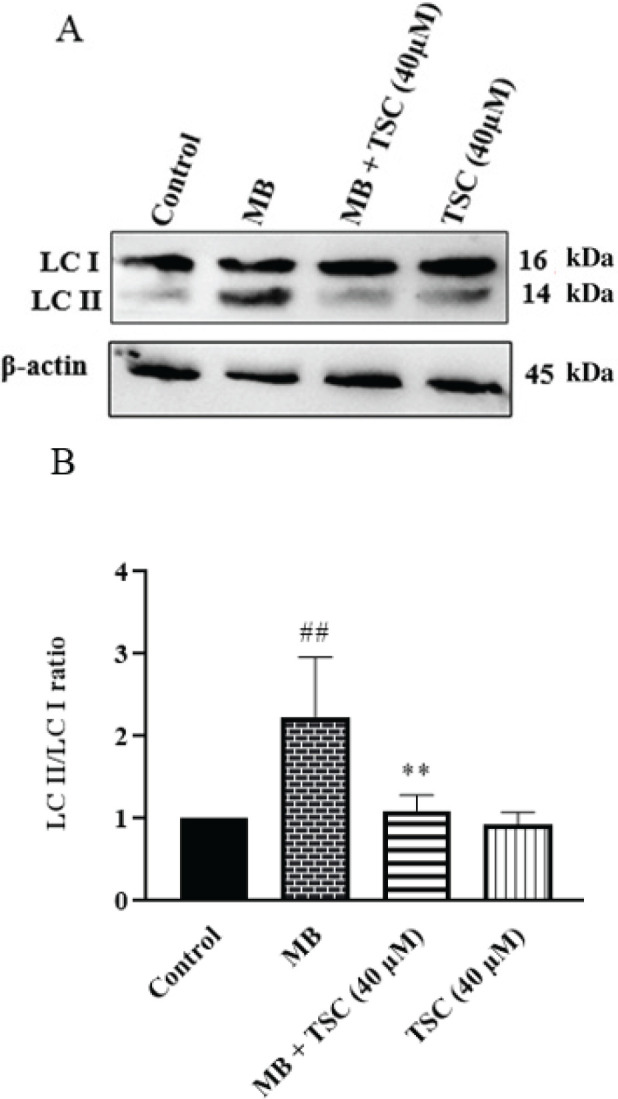
Effect of MB and TSC on LC3 II and LC3 I expression in HEK-293 cells

Western blotting was done to evaluate the effect of MB and TSC on the expression of key factors of autophagy downstream. Following exposure of HEK-293 cells to MB (9 mg/ml) for 24 hr, LC3-II/I ratio (*P*<0.01) and Beclin-1 (*P*<0.05) were meaningfully enhanced compared with the control group. Pre-incubation of HEK-293 cells with TSC (40 μM, for 24 hr) resulted in a reduction in the LC3-II/I ratio (*P*<0.01) and Beclin-1 level (*P*<0.05) in comparison with the MB group (Figures 7 and 8). 

MB: Myoglobin

TSC: Trans-sodium crocetinate

A) Western blot bands of Bax, Bcl-2, and beta-actin in HEK-293 cells exposed to MB and TSC. B) Western blot analysis of Bax/Bcl-2 ratio in HEK-293 cells exposed to MB and TSC.

Data are presented as means±SD (n=4). Data were analyzed by one-way ANOVA and the Tukey-Kramer post-test. ###*P*<0.001 vs control group and ***P<0.001 vs MB group.

MB: Myoglobin; TSC: Trans-sodium crocetinate

A) Western blot bands of cleaved caspase-3 and beta-actin in HEK-293 cells exposed to MB and TSC. B) Western blot analysis of cleaved caspase-3 expression in HEK-293 cells exposed to MB and TSC. Data are presented as means±SD (n=4). Data were analyzed by one-way ANOVA and the Tukey-Kramer post-test. ##*P*<0.01 vs control group and *P<0.05 vs MB group

MB: Myoglobin; TSC: Trans-sodium crocetinate

A) Western blot bands of Beclin-1 and beta-actin in HEK-293 cells exposed to MB and TSC. B) Western blot analysis of Beclin-1 expression in HEK-293 cells exposed to MB and TSC. Data are presented as means±SD (n=4). Data were analyzed by one-way ANOVA and the Tukey-Kramer post-test. #*P*<0.05 vs control group and *P<0.05 vs MB group.

MB: Myoglobin; TSC: Trans-sodium crocetinate.

A) Western blot bands of LC3 I, LC3 II, and beta-actin in HEK-293 cells exposed to MB and TSC. B) Western blot analysis of LC3 II/I ratio in HEK-293 cells exposed to MB and TSC. Data are presented as means±SD (n=4). Data were analyzed using one-way ANOVA and the Tukey-Kramer post-test. ##*P*<0.01 vs control group and **P<0.01 vs MB group.

MB: Myoglobin; TSC: Trans-sodium crocetinate

**Figure 9:**
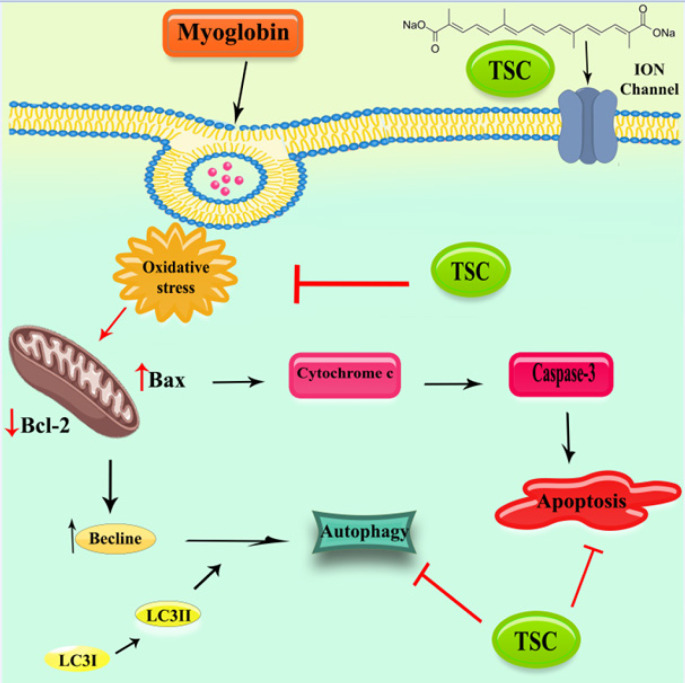
Protective effect of TSC on MB-induced cytotoxicity in HEK-293 cells via oxidative stress, autophagy, and apoptosis signaling pathways

TSC: Trans-sodium crocetinate

MB: Myoglobin; Trans-sodium crocetinate

## Discussion

The present research assessed the protective effect of TSC on MB-induced cytotoxicity through numerous signaling pathways in HEK-293 cells. According to our results, exposure of HEK-293 cells to MB for 24 hr decreased viability and increased ROS production. Furthermore, MB enhanced the apoptosis and autophagy markers in cells. However, pretreatment of HEK-293 cells with TSC for 24 hr reduced the cytotoxicity and ROS production caused by MB. Additionally, TSC pretreatment diminished the levels of autophagy and apoptosis markers in response to MB cytotoxicity. 

RM is a term used to describe the breakdown of skeletal muscles. RM leads to the release of cellular contents including enzymes, MB, and electrolytes into the extracellular fluid (32). MB is an oxygen-binding hemoprotein that is released from myocytes, and it is the main toxic factor that can result in renal injury. The development of oxidative stress is the main reason for RM-induced AKI (7). Recently, the incidence of RM has risen. Hence, the management of AKI as a deadly complication of RM is very necessary (1). Herbal medicines such as saffron can have an impact on the treatment of RM-induced AKI (26).

The* in vivo* model to study RM-induced AKI is focused on glycerol-induced AKI (7). On the other hand*,* *in vitro* studies are established to assess the direct cytotoxic effects of MB on HEK-293 cells (3). *In vitro* investigations have exhibited that cell viability was reduced in HEK-293 cells that were exposed to 200 µM MB for 24 hr (3). Furthermore, Chen *et al*., observed that MB reduced cell viability in HEK-293 cells (7). In the present study, MB at concentrations of 0 –15 mg/ml for 24 hr reduced the HEK-293 cells’ viability concentration-dependently and the IC_50_ value of MB was 9.19 ± 0.02 mg/ml.

TSC is an effective anti-oxidant that reduces intracellular ROS (33). It enhances the activity of the internal anti-oxidant enzymes and clears free radicals (23). In a previous study, pre-treatment of bovine aortic endothelial cells with crocetin (1 M) for 12 hr revealed that it reduced ROS formation (20). Additionally, crocetin (0.1, 0.5, and 1 μM, for 24 hr) was found to enhance anti-oxidant enzymes, leading to the detoxification of free radicals in cyclosporine A-mediated cytotoxicity in HEK-293 cells (23). According to previous research projects, we realize that oxidative stress has a basic and important role in MB toxicity in HEK-293 cells. Overall, our results confirmed the previous studies that pretreatment of HEK-293 cells with TSC (2.5, 5, 10, 20, 40, 80, and 100 µM) for 24 hr markedly increased cell viability and reduced the formation of ROS. 

Exposure to MB causes oxidative stress-mediated cellular damage, which can reduce anti-oxidant enzyme activity and anti-oxidant levels. Fenton reaction produces hydroxyl radicals and induces significant damage in renal cells (34). In our investigation, following exposure of HEK-293 cells to MB (9 mg/ml) for 24 hr, ROS generation was enhanced. Our findings support prior research indicating oxidative damage is a primary cause of cell death in MB-induced cytotoxic HEK-293 cells (7).

The evidence suggested that RM-induced AKI occurs through several molecular signal pathways. Among these, more evidence exhibited that apoptosis and oxidative stress were most implicated in MB-induced cytotoxicity (35). In one of these studies, MB (200 µm) elevated protein expression of caspase-9 and cytochrome C but not caspase-8 after 24 hr exposure. These findings indicated that the intrinsic pathway plays a main role in MB-induced HEK-293 cell apoptosis (3). In agreement with prior reports, our data displayed that MB triggered the overexpression of Bax and cleaved caspase-3, along with reduced Bcl-2 levels in HEK-293 cells after 24 hr.

Many studies have shown the protective role of crocetin against cellular apoptosis. Crocetin increases anti-oxidant activity and adjusts intracellular homeostasis in vascular endothelial cells (20). Additionally, crocetin increases intrinsic mitochondrial toleration against apoptotic motives in cyclosporine A-mediated toxicity in HEK-293 cells (23) and fixes the potential of the mitochondrial membrane against radiation-induced damage in intestinal epithelial cells (36).

Crocetin was found to protect against arsenic trioxide-induced kidney damage by diminishing apoptosis in rats, according to Liu *et al*. Crocetin inhibited caspase-3 and Bax while increased the level of Bcl-2 (37). Furthermore, several studies have displayed that TSC can modulate the expression of apoptotic proteins (17). In one of these investigations, TSC (1 and 5 M) for 48 hr lowered cleaved caspase-3 and the level of Bax/Bcl-2 ratio in HEK-293 cells exposed to contrast media (16). In another investigation, myocardial ischemia/reperfusion injury increased apoptosis in the heart, despite TSC treatment showing cardioprotective effects via anti-apoptosis actions (38). Consistent with previous results, in this study, TSC (40 μM, for 24 hr) pre-incubation of HEK-293 cells reduced cleaved caspase-3 and the level of Bax/Bcl-2 ratio.

Autophagy is a known self-digesting reply to lysosome-mediated degradation of injured cellular organelles or proteins and recycles nutrients for cell survivorship (39). According to research projects, in AKI, the fast and main function of autophagy has a protective figure in renal cells (40). After MB exposure, proteins and organelles are injured, and the lysosomes are increased, as a result, autophagy is stimulated and may ameliorate renal cell survivorship (7).

Research exhibited that meglumine diatrizoate (100 mg/ml) elevated the interpretation markers of autophagy in HEK-293 cells (41). Similarly, in an *in vitro* study the LC3 II/LC3 I ratio was enhanced after HEK-293 cells were exposed to 200 mg/ml of iohexol for 6 hr (42). Our findings were in line with the former research that MB (9 mg/ml) for 24 hr enhanced the LC3 II/I ratio and Beclin-1 expression in HEK-293 cells.

Crocetin demonstrated beneficial effects in breast cancer cells (MCF-7) by regulating the autophagy markers (reduced Beclin-1 level) (15). Also, according to the research by Rajabian *et al.*, TSC diminished autophagy marker levels (LC3II/I ratio and Beclin-1 level) in HEK-293 cells exposed to contrast media (16). Our finding suggested that TSC pre-treatment (40 μM, for 24 hr) reduced the Beclin-1 level and the LC3 II/I ratio in HEK-293 cells, which supported previous studies linked to TSC-regulated autophagy. 

## Conclusion

Our study demonstrates that TSC can protect HEK-293 cells from MB-induced cytotoxicity. MB causes cytotoxicity in HEK-293 cells by increasing oxidative stress. Furthermore, MB increases apoptosis (as evidenced by changes in Bax/Bcl-2 ratio and cleaved caspase-3 level) and autophagy markers (as evidenced by changes in Beclin-1 level and LC3 II/I ratio). TSC protects against MB-induced cytotoxicity via anti-oxidant characteristics, autophagy pathway regulation, and anti-apoptotic properties (as depicted in Figure 9). 

## Authors’ Contributions

H H and S M were supervisors, designed the work, revised it critically for important intellectual content, and approved the version to be published. T AF did the experiment, analyzed the data, and wrote the manuscript, A KR, M GR, and F R helped in doing the research. All authors have read and approved the paper.

## Declarations

The data that support the findings of this study are available from the corresponding author upon reasonable request.

## Ethics Approval and Consent to Participate

Not applicable

## Conflicts of Interest

The authors declare that they have no conflicts of interest.
